# Editorial: Novel technologies for soybean improvement

**DOI:** 10.3389/fpls.2022.1047739

**Published:** 2022-10-17

**Authors:** Xianzhong Feng, Deyue Yu, Madan K. Bhattacharyya

**Affiliations:** ^1^ Key Laboratory of Soybean Molecular Design Breeding, Northeast Institute of Geography and Agroecology, Chinese Academy of Sciences, Changchun, China; ^2^ National Key Laboratory of Crop Genetics and Germplasm Enhancement, National Center for Soybean Improvement, Nanjing Agricultural University, Nanjing, China; ^3^ Department of Agronomy, Iowa State University, Ames, IA, United States

**Keywords:** editorial, novel technologies, for, soybean, improvement

## Introduction

Growing human population has put enormous pressure on the global food security. In the past few decades, the soybean yield has remained stagnant especially due to the use of conventional breeding technologies. In this regard, the research community were looking for the novel breeding technologies to revolutionize the soybean breeding. “Novel breeding technologies” are biotech-based approaches to modify plant characteristics fast and accurately. In this scenario, the current Research Topic “novel technologies for soybean improvement” is intended to collect articles on recent advances and future applications of the novel technologies in the soybean breeding to boost the soybean production. These approaches and technologies include molecular design breeding approaches, genome editing and transformation technology, RNA interference approach, Marker-Assisted and Genomics-Selection breeding approaches, machine learning and bioinformatics technology. Below we briefly highlight the applications and potential of the new approaches in the soybean breeding; and how they can be significant for increasing the yield and quality in soybean that are presented in a collection of 20 papers published in the special issue on the Research Topic: Novel Technologies for Soybean Improvement.

## Computation technology has been put to use widely for soybean improvement

Soybean flower and pod drop are important determinants of soybean yield, thus use of advanced techniques that will increase the accuracy and speed for the flower and pod phenotyping has great influence on soybean breeding. In this regard, the computer vision techniques have recently emerged to phenotyping the flowers and pods in bulk at higher speed and accuracy. Zhu et al. (2022) identified that among the various deep learning algorithms, the Faster R-CNN model performance was the best to phenotype soybean flowers and pods. The accuracy of Faster R-CNN model was 94.36 and 91% for detecting the flowers and pods, respectively. Furthermore, based on the Faster R-CNN model, they also proposed the fusion model for soybean flower and pod recognition and counting, that will greatly reduce labor intensity and improve efficiency.

Identification of the soybean varieties with superior nutritional value and composition is an important goal of soybean researchers. In this regard, Wei et al. (2022) checked the performance of Terahertz frequency-domain spectroscopy and chemometrics for the identification of soybean varieties, and subsequently proposed the grey wolf optimizer-support vector machine (GWO-SVM) soybean variety identification model. These authors showed that combination of the Terahertz frequency-domain spectroscopy and GWO-SVM will identify the soybean varieties at higher accuracy and speed. Compared with discriminant partial least squares (DPLS) and particles swarm optimization support vector machine, GWO-SVM combined with the second derivative could establish a better soybean variety identification model.

Soybean possessing narrow genetic base makes it hard to screen the available genetic and phenotypic variability for identifying the superior genotypes. In this regard, “Artificial Neural Network” (ANN) is the type of machine learning process which is used to classify the narrow-range and pure lineage populations. Amaral et al. (2022) argue that ANN can be used to categorize soybean genotypes from a population that has an either a wide genetic variability or a narrow genetic variability for the relative maturity character. The authors further demonstrated that ANN can be used to discriminate genotypes in early breeding generations. This would make it easier and to rapidly find high-performing cultivars with longer and shorter photoperiod amplitudes at a single selection site.

Artificial intelligence (AI) technology has been expected to provide the accurate phenotypic data at high resolution and low cost in the plants. Xing et al. (2022) used the computer vision (CV), machine learning based technology for the phenotypic analysis in soybean. They focused on expanding four different kinds of features disease traits, indoor test traits, field traits and soybean seed phenotypic traits. It was revealed that CV technology has the ability to collect highly accurate characteristics, and allows to provide large quantity of big data for breeding programs.

## More and more novel technologies of both phonemics, genomics and bioinformatics have been applied in soybean improvement

Availability of high-throughput phenotyping systems has provided the opportunity to have an easy access to growth data. This will in turn allow to predict growth of cultivars with unknown performance by conducting the genomic prediction analysis. Till now, the genomic and growth modeling prediction have not been studied to the greater depth. In this regard, Toda et al. (2022) used the data collected by an Unmanned Aerial Vehicle (UAV) to predict genomic green fraction dynamics in soybean. In their study, the use of UAV remote sensing allowed the measurement of the longitudinal variations in the green fractions. Their study showed that the model effectively combined early growth data with training population phenotypic data for prediction. This prediction method could be applied to selection at an early growth stage in crop breeding, and could reduce the cost and time of field trials.

Salinity is one of the major environmental constraints effecting the crop yield. However, there is considerable lack of high throughput phenomics platforms that allow to record the salt stress responses of plants as well as the non-destructive collection method for root phenotypic data. Zhang et al. (2022) reported the high-throughput and low-cost phenotypic platform that allows phenotyping whole plant including roots at uniform and controlled soil stress conditions. They concluded that responses of cotyledons can be used as non-destructive indicator for determining the salt tolerance of the seedlings based on their high-throughput multiple-phenotypic assays of 178 soybean cultivars.

Photosynthesis is an important process that determines the plant growth and yield. Hence, it is prerequisite to more efficiently assay the photosynthetic capacity of crop plants in order to select the varieties with higher photosynthetic efficiency. Shamim et al. (2022) explored the underlying physiological mechanisms and genetic basis regulating the photosynthesis in the Japanese soybean germplasm by using the “MIC-100” system (high-throughput system for measuring the photosynthesis). They identified the signification association of single nucleotide polymorphism (SNPs) on Chromosome 17 with the light-saturated photosynthesis (*A*sat) trait based on genome wide association study (GWAS) analysis. The G protein alpha subunit 1 (GPA1) was identified as the strong candidate *A*sat gene. This study provides strong evidence to apply GWAS of plant germplasm for exploring the maximum potential of photosynthesis in soybean improvement.

Limited studies have been conducted to determine the molecular mechanism underlying the seed oil and starch content in soybean. These traits are important traits determining the yield and quality of soybean. Cheng et al. (2021) used an integrated transcriptomic approach for investigating the species-specific, starch-related, carbon metabolism-related and acyl-lipid-related genes in soybean and chickpea. They identified seven soybean-specific gene expression patterns, four of which are highly expressed at the middle- and late oil accumulation stages. They proposed the difference of metabolism pathways in seed oil and starch content between soybean and chickpea, this study has opened up the possibility of engineering other legumes for enhanced oil contents.

Transcriptome sequencing of full-length transcripts by long sequencing read method is an efficient approach to reveal detailed information about the events at transcriptional or post-transcriptional levels. But this approach has not been used in the soybean till now. In this context, Huang et al. (2022) used this approach to compare full-length transcriptomes in soybean genotype 09-138 infected with either soybean cyst nematode (*Heterodera glycines*) race 4 or race 5 that are avirulent and virulent, respectively to the soybean genotype. This study provides the insights about the soybean-nematode interaction which will serve as the basis for future research.

Seed germination is regulated by the hypocotyl elongation, and it determines the vitality of seedlings; however, the mechanism underlying the hypocotyl elongation has remained largely unexplored. Shen and Chen (2022) used the weighted gene co-expression network analysis (WGCNA) for the first time to understand the global regulatory network of gene expression involved in soybean hypocotyl elongation and identified a crucial regulatory module. They reported two regulatory modules. GmPRE6s-EXPANSINs submodule and GmPIF1/GmPIF3-GmSAUR1/23-EXPANSINs submodule. This study provides important genes for developing molecular markers for breeding for soybean with enhanced hypocotyl length and seed vigor.

Role of the miRNAs in regulating biological processes as well as biotic and abiotic stress responses in plants has been well documented. Hence, identification of the miRNAs is essential to determine their role in the biological processes as well as for plant responses to environmental stresses in plants. He et al. (2021) developed a highly sensitive approach for quantitatively detecting miRNAs by ligating ribonucleotide-modified DNA probes. This approach eliminates the use of complex reverse transcription-based quantitative approach. In the devised test, miR156b immediately hybridized two ribonucleotide-modified DNA probes, and amplification using universal primers followed the ligation process. The target miRNA could be detected at a 0.0001 amol level, and variations of a single base between miR156 family members could be recognized. The suggested quantitative technique works for overexpression-based genetically modified (GM) soybean. Ligation-based quantitative polymerase chain reaction (PCR) may be used to study miRNAs and investigate GM organisms.

In plants, the F-box gene family is one of the big gene families. F-box genes are involved in the regulation of plant growth as well as response to multiple environmental stresses, both biotic and abiotic. Xu et al. (2022) identified the F-box gene family in soybean genome using the bioinformatic approach, and also elucidated the role of F-box-like gene *GmFBL144* in adapting soybean to drought stress. They identified 507 F-box genes that were grouped into 11 subfamilies. The *GmFBL144* showed higher expression in the roots and ectopic expression of the *GmFBL144* in Arabidopsis enhanced the sensitivity to drought stress. Their study suggested that GmFBL144 protein negatively regulates drought stress through its interaction with small heat shock protein.

## Approaches of genome-wide analysis are becoming the mainstream for soybean gene function study

To breed soybean varieties with high seed protein, it is a prerequisite to elucidate the genetic basis of high seed protein. Qin et al. (2022) used the combined approach of linkage mapping and genome-wide association studies (GWAS) to identify the QTLs and genes underlying the seed protein in soybean. They identified major QTLs mapped to Chromosomes 6 and 20 in two segregating populations. Their study revealed the similar performance of both BL and rrBLUP, and the accuracy of GS was dependent on size of training population and SNP set. The protein-specific SNPs showed higher GS efficiency compared to that by all SNPs.

The three-seeded pod number is an important trait that positively influences soybean yield. Soybean variety with increased three-seeded pod number contributes to the seed number/plant and higher yield. The candidate genes of the three-seeded pod may be the key for improving soybean yield. In this regard, Li et al. (2021) identified and validated the candidate genes regulating the three-seeded pod. They used the QTL mapping approach and identified 36 QTL. Four genes from 162 genes underlying these two QTL related to pod and seed set were identified as potential candidate genes controlling this trait. These four genes can be used as molecular markers for incorporating this trait into commercial soybean cultivars.

## Genome editing and transformation technology are reaching to maturity to reap benefits for soybean improvement

In the past four decades the genetic transformation was used to modify the genome of soybean for the trait improvement. However, the genetic transformation in soybean is still subjected to low transformation efficiency, genotype specificity as well as long and tedious transformation processes. Xu et al. (2022) reviewed the genetic transformation progress in soybean and reported the gain made in efficiency and genotype flexibility of soybean transformation over the last decade. Factors that influence the soybean transformation are proper explant selection, reagent selection, components of culture medium and the detailed understanding of the mechanism involved in the transformation.

CRISPR/Cas9 genome editing has emerged an efficient technique to breeding crop cultivars with enhance tolerance to environmental stresses. In Arabidopsis, knockout of the entire family of the abscisic acid (ABA)-induced transcription repressors (AITRs) enhanced drought and salinity tolerance without any fitness costs. Wang et al. (2021) demonstrated that mutation of *GmAITR* genes by CRISPR/Cas9 genome editing in soybean increased the salinity tolerance. The enhanced salt tolerance was observed both at the seed germination and seedlings stages of *gmaitr* mutants. Hence, the knockout of the *GmAITR* genes *via* CRISPR/Cas9 proved to be a powerful approach for inducing salt tolerance in soybean.

Insect pests such as leaf-chewing insects are one of the major constraints in soybean production. In this context, the development of soybean varieties with increased resistance to leaf-chewing insects can greatly increase the soybean yield. The *Glyma.07g110300* (*GmUGT*) encodes an UDP-glycosyltransferase (UGT) which is the core enzyme that negatively regulates insect resistance. Zhang et al. (2022) demonstrated that CRISPR/Cas9-mediated mutagenesis/knockout of *GmUGT* enhanced soybean resistance to *Helicoverpa armigera* and *Spodoptera litura*. The ectopic expression of the *GmUGT* gene in Arabidopsis *ugt72b1* mutant this mutant significantly reduced its resistance to *H. armigera*.

Soybean mosaic virus (SMV) is a prevalent soybean pathogen. Pyramiding multiple SMV-resistance genes into one individual is tedious and difficult. Targeting the viral genome *via* host-induced gene silencing (HIGS) has potential to induce broad-spectrum resistance (BSR) to SMV. Jiang et al. (2021) employed the *Nicotiana benthamiana*-soybean mosaic virus (SMV) system to optimize the target SMV sequence for HIGS. They were able to use the information gained from the *N. benthamiana*-SMV system to develop transgenic soybean lines with effective HIGS to enhance SMV resistance in soybean.

*Florigen* is a key player regulating the balance between vegetative and reproductive growth. Hence, it regulates the crop yield; but the actual utilization of the *florigen* in improving the crop yield remains largely unexplored. In this context, Xu et al. (2021) developed a strategy to improve the yield of soybean significantly by using the florigen. They showed that fine-tuning of the *florigen* genes *via* RNAi can boosts soybean yield. The conserved functions of *florigen* in plants and use of the RNAi approach to manipulate the expression of this gene therefore may be used to increase crop productivity.

To maintain the crop germplasm for its use in future breeding programs, preservation of the pollen grains *via* ultra-low temperature approach is an effective and safe way for long-term storage of plant germplasm resources. Jia et al. (2022) reported an efficient ultra-low temperature freezing approach to preserve soybean pollens. Soybean flowers collected at the fully-bloom stage were dried, frozen and stored at -196 or -80°C. They observed that 90% of the pollen grains remain viable based on *in vitro* tests. Their study documented that this technique of pollen preservation will break the spatiotemporal barrier of soybean hybridization and facilitate efficient soybean breeding.

## Author contributions

XF wrote the draft, DY and MB edited. All authors contributed to the article and approved the submitted version.

## Funding

This research was funded by National Nature Science Foundation of China (No. U21A20215).

## Conflict of interest

The authors declare that the research was conducted in the absence of any commercial or financial relationships that could be construed as a potential conflict of interest.

## Publisher’s note

All claims expressed in this article are solely those of the authors and do not necessarily represent those of their affiliated organizations, or those of the publisher, the editors and the reviewers. Any product that may be evaluated in this article, or claim that may be made by its manufacturer, is not guaranteed or endorsed by the publisher.

